# Prevalence of Ophthalmological Manifestations in Patients with Inborn Errors of Immunity: A Systematic Review and Meta-Analysis

**DOI:** 10.1007/s10875-025-01880-4

**Published:** 2025-05-13

**Authors:** Laura Zárate-Pinzón, Germán Mejía-Salgado, Carlos Cifuentes-González, Oscar Correa-Jiménez, Stefania Amaris, Alberto Alfaro-Murillo, Juanita Téllez-Zambrano, Angie Verbel, Paula Monje-Tobar, Alejandra de-la-Torre

**Affiliations:** 1 Ophthalmology Interest Group Universidad del Rosario (OIG UR), Escuela de Medicina y Ciencias de la Salud, Universidad del Rosario, Bogotá, Colombia; 2 Neuroscience Research Group (NEUROS), Neurovitae Center for Neuroscience, Institute of Translational Medicine (IMT), School of Medicine and Health Sciences, Universidad del Rosario, Bogotá, Colombia; 3 Pulmonology and Immunology in Pediatrics Research Group, Department of Pediatrics, School of Medicine, Universidad Nacional de Colombia, Bogotá, Colombia; 4 Division of Clinical Immunology, Department of Internal Medicine, Hospital San Juan de Dios-Caja Costarricense de Seguro Social, San José, Costa Rica; 5 Neuroscience Research Group– NeURos, Escuela de Medicina y Ciencias de la Salud, Universidad del Rosario, Carrera 24 # 63C 69, Bogotá, Colombia; 6 Centre of Excellence in Ocular Inflammation, Colombian Visual Science and Translational Eye Research Institute (CERI), Bogotá, Colombia

**Keywords:** Primary immunodeficiency diseases, Eye disease, Ocular manifestations, Prevalence, Inborn errors of immunity, Meta-analysis

## Abstract

**Background:**

Although some reports indicate ocular involvement in Inborn Errors of Immunity (IEI) patients, the characteristics of this association remain unclear. Increased awareness can facilitate early diagnosis and prevention of visual complications.

**Objective:**

To determine the prevalence and characterize ophthalmological manifestations in patients with IEI.

**Methods:**

A systematic literature search was performed across Embase, PubMed, and Lilacs. Observational studies with at least 10 IEI patients exhibiting ophthalmological manifestations were reviewed. A meta-analysis using a random effects model, weighted proportion, and 95% confidence intervals were reported as appropriate.

**Results:**

Sixty-two articles out of the 6,884 studies were included. The pooled prevalence of ocular manifestations in IEI patients was 54% (95%CI = 39–69), with a mean age of 11.1 ± 7.8 years and male predominance. Regarding the type of IEI with ocular involvement, the most frequently affected group was the Combined immunodeficiencies with associated or syndromic features (82%, 95%CI = 66–91), followed by the diseases of immune dysregulation (73%, 95%CI = 27–95), auto-inflammatory disorders (48%, 95%CI = 10–88), and congenital defects of phagocytes (39%, 95%CI = 11–76). Europe had the highest prevalence of patients with ocular manifestations (68%, 95%CI = 32–90). The most common ocular manifestations observed in IEI patients were those affecting ocular mobility, followed by those that involved the anterior segment, posterior segment, eyelids, and adnexal structures.

**Conclusions:**

These results highlight a significant burden of ocular involvement in IEI patients, mainly during childhood and associated with amblyogenic factors. Therefore, ophthalmologists, pediatricians, and immunologists must be involved in early detection to prevent ocular complications and overall well-being.

**Supplementary Information:**

The online version contains supplementary material available at 10.1007/s10875-025-01880-4.

## Introduction

Inborn errors of immunity (IEI) are a heterogeneous group of disorders characterized by genetic defects in the development or function of the innate and adaptive immune pathways [1–3]. Clinically, these diseases manifest with a wide range of features, including enhanced susceptibility to infections, heightened severity of infections, predispositions to autoimmunity, autoinflammatory conditions, allergies, malignancies, and other aberrant immune responses [1–4].

The eye is an immune-privileged organ with self-regulatory immune mechanisms to preserve vision, such as anatomical and cellular barriers, eye-derived immunological tolerance in the anterior chamber, and an immune-suppressive intraocular microenvironment [5, 6]. Disrupting these immune defense mechanisms can result in ocular inflammation or infections [7].

While ocular involvement in patients with IEI has been documented as relatively uncommon, it can present itself in diverse ways. Intriguingly, many of these manifestations may serve as initial symptoms, potentially guiding the diagnosis of a specific IEI [8]. It has been reported that 11.2% to 34.3% of patients with IEI have ocular manifestations [7, 9]. However, this observation is primarily derived from small case series and cross-sectional studies [7, 9]. Therefore, this study aimed to determine the prevalence and characterize ophthalmological manifestations in patients with IEI through a systematic review and meta-analysis.

## Methods

This was a systematic review and meta-analysis according to the ‘Preferred Reporting Items for Systematic Review and Meta-analysis (PRISMA) and Meta-analysis of Observational Studies in Epidemiology (MOOSE) guidelines (Supplementary material). The protocol was registered in the International Prospective Register of Systematic Reviews (PROSPERO ID number *CRD42022350159)*. No institutional review board approval was needed, as this study is based on publicly available data and did not use individual-level data.

### Search Methods and Strategy

**Fig. 1:**
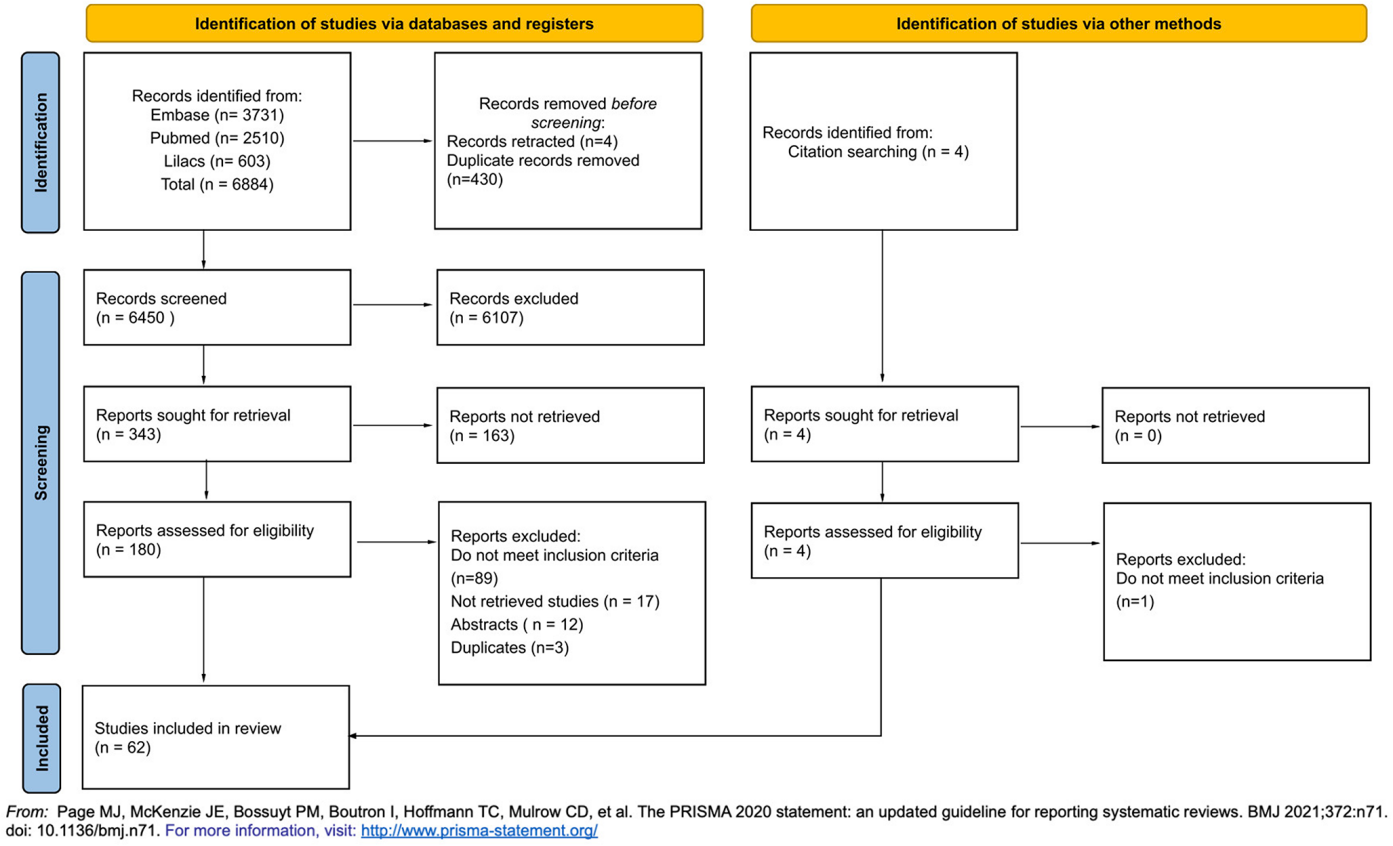
PRISMA flow chart

The search was undertaken across the following databases: Embase, PubMed, and Lilacs. The search strategy included terms reflecting the disease of interest (inborn errors of immunity or primary immunodeficiency disease) and ocular manifestations (eye manifestations). The search strategies were modified to meet the criteria of each database **(Supplementary material)** and were done on March 21, 2022. This process was documented following the PRISMA statement and is available in Fig. 1.

### Study Eligibility Criteria

Primary observational studies with a minimum of 10 patients diagnosed with IEI were included, encompassing case series, case-control studies, cohort studies, cross-sectional studies, and clinical trials. No language restriction was applied. Excluded from consideration were non-full-text articles, case series involving fewer than ten patients, studies conducted in species other than humans, case reports, economic analyses, systematic reviews, and secondary data sources.

### Patient Inclusion and Exclusion Criteria

Studies that reported on patients of all ethnicities, ages, and genders with IEI who exhibited any ophthalmological manifestation were included. Conversely, we excluded studies that involved patients in whom IEI could not be confirmed.

### Study Selection

All search results were imported in RIS format and cataloged using Zotero^®^ reference management software, forming an integrated database of sourced articles. This is followed by a thorough examination for duplicated entries within Zotero^®^ and subsequent verification in Microsoft Excel^®^, emphasizing authors’ names, publication titles, and DOIs. After eliminating duplicates, the remaining titles and abstracts were systematically distributed and independently assessed by three pairs of evaluators: CHCG-LZP, GAMS-LPP, and SA-OCJ. Concordance among groups ranges from 83% to 89%. This double-step assessment procedure entailed scrutinizing titles and abstracts in alignment with the pre-established inclusion and exclusion parameters.

After this assessment, articles were classified as “included,” “excluded,” or “ambiguous” in a Microsoft Excel^®^ spreadsheet. Any discrepancies encountered during the paired assessment were meticulously addressed through collaborative deliberation among authors. When the mutual agreement was challenging, guidance from two immunologist experts was enlisted to finalize the selection, ensuring the integrity and uniformity of the article inclusion process.

### Data Extraction

Data were extracted by eight independent investigators (SA, PAMT, OCJ, LZP, JTZ, GAMS, PL, AV) using a standardized and validated form in Microsoft Excel^®^. The following characteristics were extracted from each eligible study: (1) the author’s name, (2) the article title, (3) DOI, (4) the year of publication, (5) study sites/locations, (6) study methodology, (7) sampling size, (8) age, (9) gender; (10) patients affected with IEI, (11) type of IEI according to the latest International Union of Immunological Societies (IUIS) classification on Inborn Errors of Immunity, (12) patients affected with IEI with ocular manifestations, (13) type of IEI of patients affected with ocular manifestations, (14) type of ocular manifestations.

### Risk of Bias Assessment

The risk of bias assessment was conducted using validated tools depending on the methodological design of the article. For cohort and case-control studies, we used the Clinical Advances Through Research and Information Translation (CLARITY) device, which McMaster University contributed [10, 11]. Cohort studies were assessed on (1) selection of exposed and non-exposed cohorts, (2) assessment of exposure, (3) outcome of interest not present at the start of the study, (4) exposed and unexposed matching, (5) prognostic factors, (6) assessment of outcome, (7) follow up, and (8) co-interventions. Case-control studies were assessed on (1) assessment of exposure, (2) ascertainment of exposure, (3) selection of cases, (4) selection of controls, and (5) comparability and analysis of the data.

Cross-sectional studies were assessed using the Hoy et al. modified tool for evaluating the risk of bias in prevalence studies [12]. This tool consists of 10 items addressing four domains plus a summary risk of bias assessment, which are 1) the study’s target population and 2) sampling frame representation, 3) sample selection, 4) likelihood of nonresponse, 5) data collection source, 6) case definition, 7) parameters measurement, 8) data collection consistency, 9) follow up period, and 10) appropriateness of numerator and denominator for the parameter of interest; the four domains are selection, nonresponse, measurement and analysis bias. Finally, case series studies were assessed with Hassan Murad’s Methodological quality and synthesis of case series and case reports’ assessment scale [13]; estimated (1) population selection, (2) ascertainment of exposure or outcome, (3) causality, and (4) sufficient reporting details.

In cohort and case-control studies, to simplify the scoring (Definitely yes; Probably yes; Probably no; Definitely no), we assigned the following values: if the question was answered with ‘Definitely yes,’ we assigned ‘low risk of bias’; if was ‘Probably yes’ or ‘Probably no,’ we assigned ‘some concern’; if was ‘Definitely no,’ we assigned a ‘high risk of bias.’

For cross-sectional studies, all ‘yes’ accounts for one point; the external validity was labeled ‘High’ for scores 0–1, ‘Some Concerns’ for scores 2, and ‘Low’ for scores 3. Similarly, internal validity was assessed as ‘High’ for scores 0–2, ‘Some Concerns’ for three, and ‘Low’ for four. Ultimately, studies were considered to have a ‘high risk of bias’ if any domains (internal or external validity) or questions for CLARITY received a ‘high risk of bias’ rating.

In the case of series studies, to simplify the scoring, we assigned the following values: if the question was answered ‘yes,’ we assigned a ‘low risk of bias’; if it was answered ‘no,’ we assigned a ‘high risk of bias,’ and if was not applicable we assigned ‘some concerns.’ The figures were generated using the Robvis tool [14].

### Data Synthesis and Statistical Analysis

Statistical analyses were performed using R version 4.3.1 (R Foundation for Statistical Computing, Vienna, Austria). Proportional meta-analyses with 95% confidence intervals (CI) were conducted to generate forest plots for categorical data. The I2 statistic was used to assess the heterogeneity, with the following thresholds: low (I2 2 < 50%), moderate (50%–75%), and high (> 75%) [15]. A fixed-effects model would be applied if heterogeneity was low (I2 < 50%), and a random-effects model was employed if heterogeneity was significant (I2 > 50%). Additionally, subgroup analyses were conducted based on the type of IEI according to IUIS classification with ocular manifestations and the geographic region where the study was conducted. Furthermore, sensitivity analyses were conducted, including only studies of higher quality. Results were considered statistically significant if *p* < 0.05. Finally, a pooled analysis was performed in some continuous measures, as we did not have comparators.

## Results

### Search Results

The analysis identified a total of 6,884 studies. After removing duplicates and retracted articles, 6,450 articles were screened based on their title and abstract, of which 6,107 were deemed not eligible. Then, 343 articles were sought for retrieval, of which 180 articles were assessed for full-text screening. Eighty-nine articles were excluded as they did not fulfill inclusion criteria, seventeen were excluded for not-retrieval, twelve were excluded for being abstracts, and three were duplicates. Additionally, four studies were identified via other methods, of which one was excluded as it did not meet the inclusion criteria. Finally, sixty-two articles were included. The PRISMA flow chart for the search strategy is presented in Fig. 1.

### Study Characteristics

Table 1 summarizes the characteristics of the included studies. Of these, thirty-one were cross-sectional studies [7, 16–45], twenty-five were case series [46–70], five were case-control studies [71–75], and one was a cohort study [76]. Eighteen studies were conducted in the Middle East (Turkey, Saudi Arabia, Egypt, Qatar, Iran, and Israel); seventeen studies were conducted in America (United States of America (U.S.), Canada, and Brazil); fourteen studies were conducted in Europe (Italy, France, Finland, United Kingdom, Slovenia, Norway, Ukraine, and Poland); nine studies were conducted in Asia (India, China, Taiwan, Pakistan, Kuwait, Japan, and South Korea); two studies did not specify location; one study was conducted in Switzerland and U.S.; one study was conducted in Australia. Studies were published between 1968 and 2022.

**Table 1:** Characteristics of included studies

Author, Year	Country	Study Design	Cohort Size (Total patients)	Age of IEI presentation (Mean ± SD)	Sex (M/F)	Type of IEI according to IUIS classification	Patients with IEI	Patients with IEI and ocular manifestations
Yaz I, et al. 2021 [46].	Turkey	Case Series	15	10.1 ± 15.8	10/5	V	15	1
Azizi G, et al. 2020 [16].	Iran	Cross-sectional	461	NA	290/171	I	310	50
Yadav R, et al. 2020 [17].	India	Cross-sectional	90	1.4 ± 0.6	72/18	I, V	52	1
Bistritzer J, et al.2021 [47].	Israel	Case Series	15	2.66 ± 3.68	NA	II	15	2
Deepti S, et al.2021 [18].	India	Cross-sectional	107	NA	NA	VII	44	6
Sukaiti N, et al. 2021 [19].	Australia	Cross-sectional	36	NA	15/21	I	36	4
Ferre E, et al. 2016 [20].	United States of America	Cross-sectional	35	NA	14/21	II	35	4
Tunakan Dalgiç C, et al. 2021 [21].	Turkey	Cross-sectional	92	40.92 ± NA	50/42	Not specified	92	1
Luo J, et al. 2021 [48].	China	Case Series	10	1.075 ± 0.48	10/0	II	10	10
Boyarchuk O, et al. 2020 [22].	Ukraine	Cross-sectional	64	14.8 ± 7.1	34/30	II	64	49
Al-Sulaiman R, et al. 2020 [49].	Qatar	Case Series	12	NA	8/4	IV	12	2
Yeh Y, et al. 2020 [71].	Taiwan	Case-Control	29	2.5 ± 3.6	29/0	III	19	2
Barkai T, et al. 2020 [23].	Israel	Cross-sectional	16	38.5 ± 16.8	9/7	V	16	1
Qureshia S, et al. 2020 [50].	Pakistan	Case Series	43	4.2 ± 4.1	NA	I, II, IV, V, VIII	20	2
Massaad M, et al. 2020 [24].	Kuwait	Cross-sectional	286	NA	146/140	I, II, III, IV, V, VII, VIII	57	6
Faruk Incecik F, et al. 2020 [51].	Turkey	Case Series	31	3.84 ± 2.19	14/17	II	31	2
Marques I, et al. 2019 [52].	Brazil	Case Series	14	NA	5/9	IV	14	4
Papadopoulou C, et al. 2019 [53].	Multicentric	Case Series	11	7.55 ± 7.08	5/6	V, VI, VII	10	3
Esenboga S, et al. 2017 [25].	Turkey	Cross-sectional	32	NA	NA	III	32	2
Lodice A, et al. 2017 [26].	Italy	Cross-sectional	15	1.033 ± 0.33	11/4	II	15	15
Mariani L, et al. 2017 [72].	France	Case-Control	57	NA	29/28	II	17	10
Akturk H, et al. 2017 [27].	Turkey	Cross-sectional	91	1.28 ± 0.09	45/46	II	91	84
Coulter T, et al. 2017 [28].	None declared	Cross-sectional	53	NA	34/19	III	53	13
Blazing S, et al. 2016 [29].	Slovenia	Cross-sectional	247	NA	147/100	I, II, III, IV, V, VI, VII, VIII	247	5
Nanthapisal S, et al. 2016 [30].	England	Cross-sectional	15	16.4 ± 10.7	NA	VII	15	1
Patirogluh T, et al. 2016 [54].	Turkey	Case Series	20	NA	NA	IV	20	11
Salman M.S, et al. 2015 [31].	Canada	Cross-sectional	184	15 ± 7.7	92/92	II	184	115
Méneret A, et al. 2014 [73].	France	Case-Control	67	NA	NA	II	67	13
Nagai K., et al. 2013 [55].	Japan	Case Series	15	NA	7/8	IV	15	14
Greenberger S, et al. 2013 [32].	Israel	Cross-sectional	32	11.8 ± 5.4	19/13	II	32	31
Malgorzara P, et al. 2013 [56].	Poland	Case Series	33	NA	33/0	III	33	6
Shaikh A, et al. 2010 [74].	Switzerland, United States of America	Case-Control	24	NA	NA	II	13	13
Alaaeldin F, et al. 2010 [33].	Egypt	Cross-sectional	113	NA	62/51	IV	113	113
Tsilou E, et al. 2010 [34].	United States of America	Cross-sectional	196	NA	101/95	II	50	31
Shaikh A, et al. 2009 [35].	United States of America	Cross-sectional	13	NA	7/6	II	13	13
Al-Muhsen S, et al. 2009 [76].	Saudi Arabia	Cohort	32	NA	25/7	V	32	14
Khan A, et al. 2008 [57].	Saudi Arabia	Case Series	13	17.1 ± 11.9	4/7	II	11	11
Nofech-Mozes Y, et al. 2007 [36].	Canada	Cross-sectional	14	NA	NA	I	14	2
Riise R, et al. 2007 [58].	Norway	Case Series	10	NA	7/3	II	10	10
Moin M, et al. 2007 [37].	Iran	Cross-sectional	104	NA	54/50	II	104	104
Winkelstein J, et al. 2006 [38].	United States of America (multicentric)	Cross-sectional	201	NA	201/0	III	201	42
Rezaei N, et al. 2005 [59].	Iran	Case Series	26	11.04 ± 5.44	14/12	IV, V	26	2
Farr A, et al. 2002 [39].	United States of America	Cross-sectional	63	NA	27/36	II	63	57
Kivitie-Kallio S, et al. 2001 [40].	Finland	Cross-sectional	29	NA	NA	V	29	29
Boerkoel C, et al. 2000 [41].	Canada	Cross-sectional	39	NA	23/16	II	39	7
Kawame H, et al. 1998 [60].	United States of America	Case Series	18	NA	8/10	II	18	11
Lewis R, et al. 1999 [61].	United States of America	Case Series	56	NA	NA	II	56	56
Rudge P, et al. 1996 [62].	England	Case Series	13	31.5 ± 16.7	11/2	III	13	5
Ziv Y, et al. 1992 [42].	Israel	Cross-sectional	19	NA	NA	II	19	19
Palestine A, et al. 1983 [63].	United States of America	Case Series	32	NA	13/19	II	32	9
Latkany P, et al. 2002 [64].	United States of America	Case Series	16	22.8 ± 14.0	NA	V	16	16
Woods C, et al. 1992 [43].	British islands	Cross-sectional	70	NA	41/29	II	70	70
Goldblatt D, et al. 1999 [75].	England	Case-Control	74	NA	NA	V	38	9
Alyasin S, et al. 2019 [65].	Iran	Case Series	18	10.92 ± 3.24	7/11	II	18	13
Cohen L, et al. 1984 [66].	United States of America	Case Series	19	NA	NA	II	12	12
Farina L, et al. 1994 [67].	Italy	Case Series	12	NA	6/6	II	12	12
JaY B, et al. 1968 [68].	England	Case Series	62	NA	NA	II	62	62
Kim S, et al. 2003 [69].	South Korea	Case Series	17	NA	12/5	V	17	6
Veerapandiyan A, et al. 2011 [44].	United States of America (multicentric)	Cross-sectional	50	17.4 ± 17.2	30/20	II	50	20
Pham M, et al. 2022 [7].	Multicentric (United States of America and Canada)	Cross-sectional	4624	NA	2666/1958	I, II, III, IV, V, VI, VII, VIII	4624	519
Manjunath M, et al. 2020 [45].	India	Cross-sectional	100	9.04 ± 3.52	60/40	II	100	100
Huryn L, et al. 2022 [70].	United States of America	Case Series	11	30.1 ± 18.2	5/6	VII	11	11

I: Immunodeficiencies affecting cellular and humoral immunity, II: Combined immunodeficiencies (CID) with associated or syndromic features, III: Predominantly antibody deficiencies, IV: Diseases of immune dysregulation, V: Congenital defects of phagocyte, VI: Defects in intrinsic and innate immunity, VII: Auto-inflammatory disorders, VIII: Complement deficiencies, NA: Not Available

The mean age of the patients was 11.1 ± 7.80 years. The most common type of IEI, according to the IUIS classification, was Combined immunodeficiencies (CID) with associated or Syndromic Features presented in thirty-five studies, followed by Congenital defects of phagocytes in fourteen studies, Diseases of immune regulation in ten studies, Immunodeficiencies affecting cellular and humoral immunity and Predominantly Antibody Deficiencies in eight studies each, Auto-inflammatory disorders in seven studies, Complement deficiencies in four studies, and Defects in Intrinsic and Innate Immunity were presented in three studies.

### Risk of Bias Assessment

Thirty-one of the sixty-two selected articles were cross-sectional studies evaluated with the Hoy et al. modified tool, of which fourteen had a low risk of bias, six had some concern, and eleven had a high risk of bias. Twenty-five case series studies were evaluated using the Hassan Murad tool, of which twenty-two had a high risk of bias, and three had some concerns. Case-control and cohort studies were assessed using the CLARITY tool; all five Case-control studies had a low risk of bias. And the one cohort study had concerns (Figures S1-S4).

### Qualitative Synthesis

#### Ocular Mobility Compromise

Nystagmus emerged as the predominant manifestation regarding ocular mobility, reported in 18 selected studies [22, 27, 31, 35, 36, 39, 42, 45, 47, 52, 54, 57, 58, 61, 62, 67, 72, 74]. This was followed by oculomotor apraxia, documented in 13 studies [7, 22, 35, 37, 45, 51, 57, 58, 61, 65, 67, 73, 74]. Meanwhile, strabismus was present in ten studies, such as endo deviation, exo deviation, hyper deviation, and exotropia [7, 26, 31, 39, 49, 54, 57, 58, 60] (Table S1).

### Ocular Manifestations in the Eyelid and Adnexal Structures

Anomalies in the eyelids, such as high-arched or wavy eyelids, long/thick eyelashes, thick eyebrows, ptosis, short palpebral fissures, hypertelorism, epicanthal folds, hooded eyelids, and upslanting palpebral fissures were described in four studies [40, 44, 60, 68]. Hordeolum was present in two studies [7, 76]. Anomalies of the lacrimal drainage system, such as the absence of punctum, nasolacrimal, and orbital cellulitis, were each reported in one of the studies [28, 34] (Table S1).

### Ocular Manifestations in the Anterior Segment

Regarding anterior segment ocular manifestations, ocular telangiectasis was reported in twelve studies [17, 22, 27, 32, 37, 39, 45, 58, 65–67, 73], followed by conjunctivitis in ten [7, 16, 18, 25, 28, 38, 46, 53, 56, 59]. Both keratoconjunctivitis and blepharoconjunctivitis were documented in five studies each [7, 20, 29, 34, 63]. While allergic rhinoconjunctivitis was reported only in one study [29].

Corneal manifestations, including corneal opacity, corneal ulcers, superficial punctate keratitis, and herpetic keratitis, were noted in six studies [7, 18, 28, 41, 60, 68]. Cataracts were reported in five studies [7, 34, 42, 60, 64]. Meanwhile, refractive errors, specifically myopia and astigmatism, were documented in four studies [26, 34, 40, 41] (Table S1).

### Posterior Segment Ocular Involvement

Optic nerve diseases, including papilledema and optic nerve atrophy, were the predominant posterior segment manifestations noted in seven studies [7, 18, 30, 31, 41, 62, 70]. Chorioretinal scars were documented in five studies [63, 64, 69, 75, 76]. Retinal anomalies encompassing pigmentary anomalies such as cafe-au-lait macules, hypopigmented macules, and melanocytic nevi were reported in four studies [32, 34, 54, 66].

Retinitis, encompassing conditions like Cytomegalovirus (CMV) retinitis and retinitis pigmentosa, was documented in three studies [24, 48, 62]. Macular edema and glaucoma were reported in another two studies [69, 70]. Additionally, one study documented retinal pigment epithelium (RPE) atrophy and neovascular membrane [69] (Table S1).

### Other Ocular Manifestations

Ocular inflammation, such as iritis, anterior uveitis, vitritis, posterior uveitis, panuveitis, and episcleritis, was documented in eight studies [7, 18, 21, 23, 24, 53, 64, 76]. Furthermore, one study reported eye infections (*Klebsiella pneumoniae*, *Haemophilus influenzae*, *Salmonella* species) [19]. Photophobia was noted in five studies [7, 18, 31, 58, 62]. While alterations in visual fields were documented in two [31, 62]. Oculocutaneous albinism appeared in five studies [32, 33, 50, 52, 55], and one study reported anhidrosis and deafness-dystonia-optic neuronopathy (DDON) syndrome, each [70, 71] (Table S1).

### Meta-Analysis

#### Overall Prevalence of Ocular Involvement in IEI Patients

**Fig. 2:**
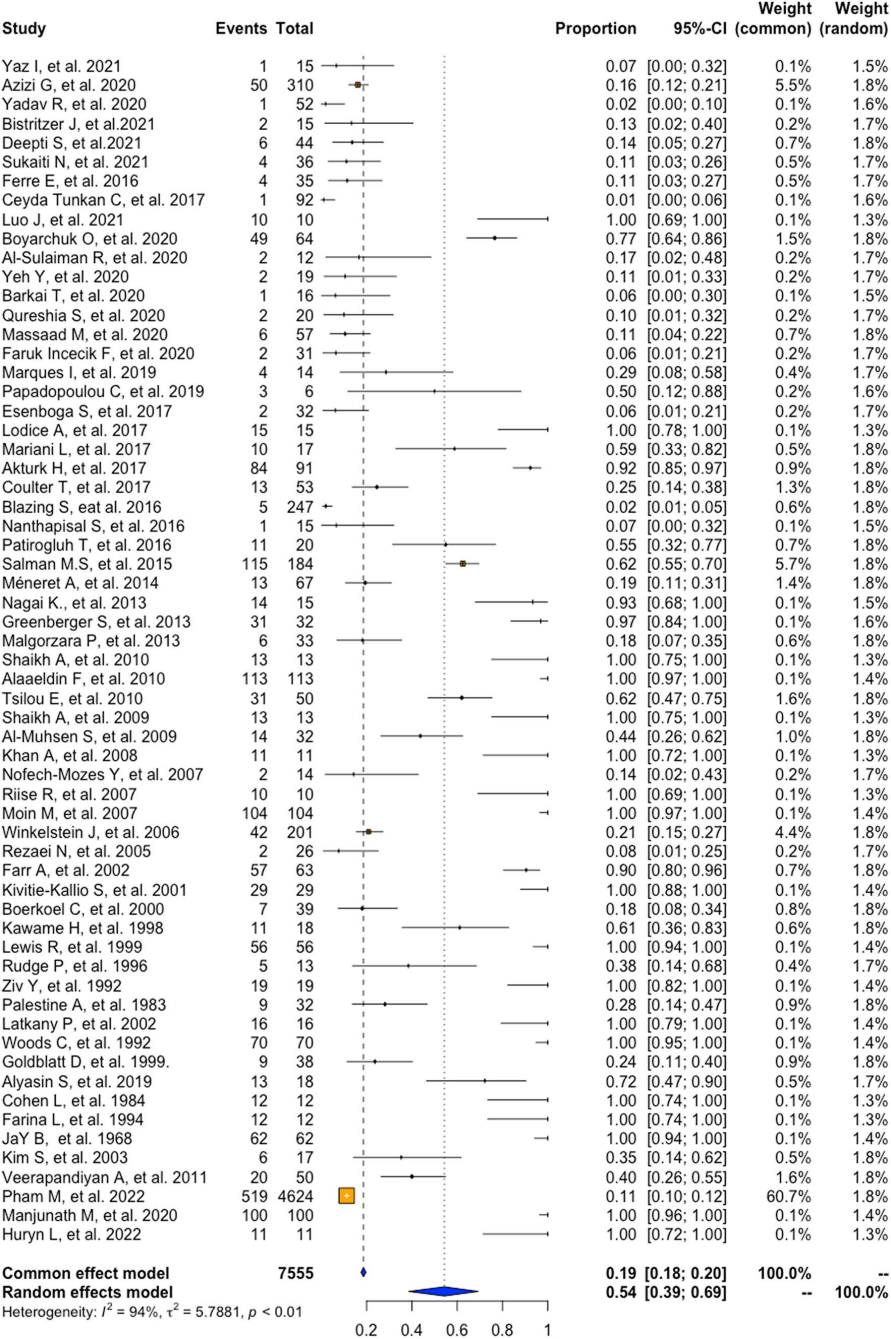
Overall prevalence of ocular manifestations in patients with IEI

**Fig. 3:**
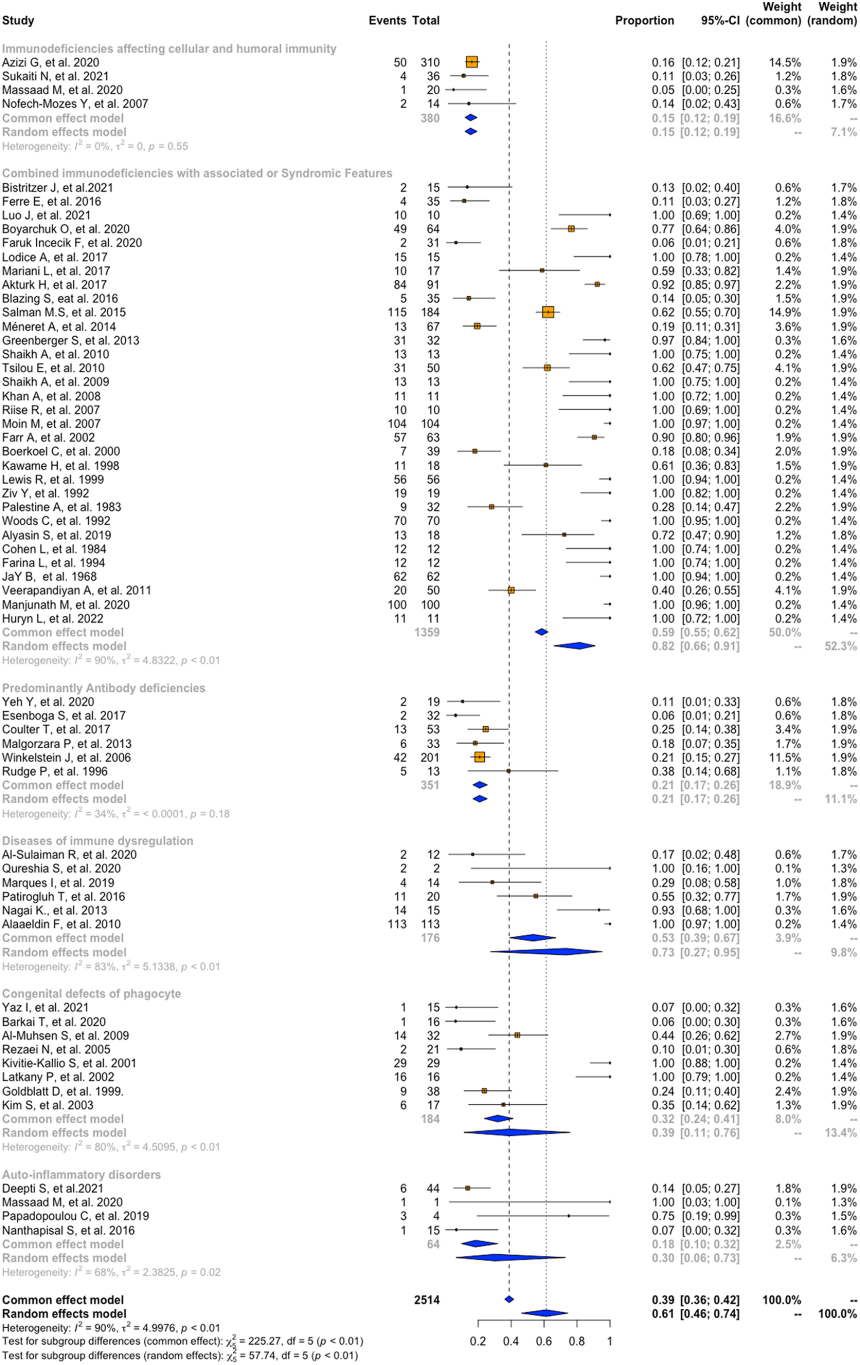
Prevalence of ocular manifestations in PIDs according to the IUIS classification

**Fig. 4:**
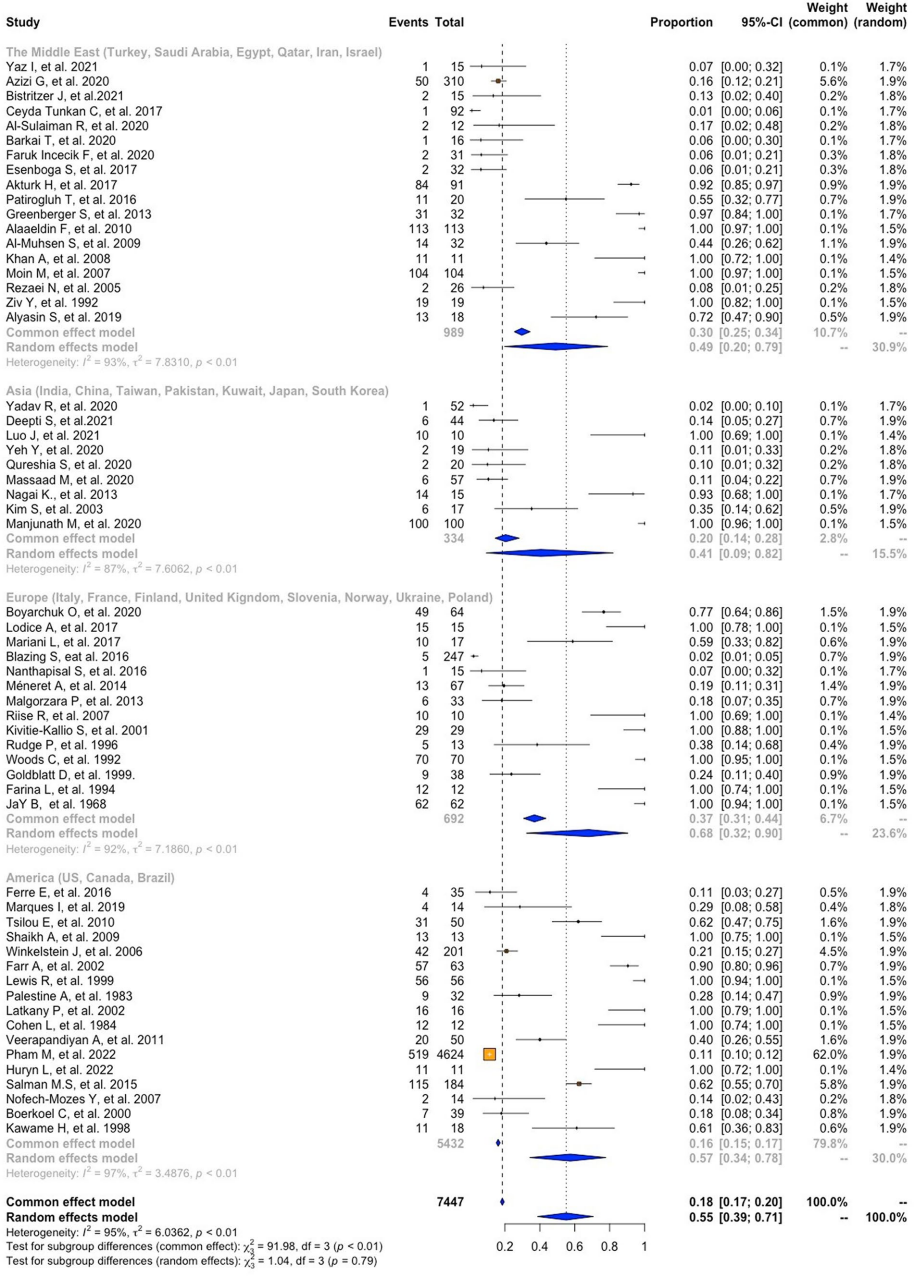
Prevalence of ocular manifestations in patients with PIDs regarding geographical localization

A meta-analysis of proportions was performed to determine the pooled prevalence of ocular manifestation in patients with IEI, shown in Figs. 2, 3 and 4. Of the sixty-two studies, the pooling results show a prevalence of 54% (95%CI = 39–69, I2 = 94%, p = < 0.01). Of the twenty-two studies that provided information on gender for 1,067 patients, 59% (95%CI = 55–63, I2 = 35%, *p* = 0.05) were male, and 41% (95%CI = 37–45, I2 = 35%, *p* = 0.05) were female (Table 2).

**Table 2:** Prevalence of ophthalmological manifestations in IEI regarding different conditions

Category	No. of Studies	No. of Patients	Pooled Incidence 95% CI	*P* of Chi-Square	I2	Selected Model
Overall prevalence	62	7555	54 (39–69)	< 0.01	94%	Random effects model
Concerning gender						
Male	22	1067	59 (55–63)	0.05	35%	Common effect model
Females	22	1067	41 (37–45)	0.05	35%	Common effect model
Concerning IUIS classification						
I. Immunodeficiencies affecting cellular and humoral immunity	4	380	15 (12–19)	0.55	0%	Common effect model
II. Combined immunodeficiencies with associated or Syndromic Features	32	1359	82(66–91)	< 0.01	90%	Random effects model
III. Predominantly Antibody deficiencies	6	351	21 (17–26)	0.18	34%	Common effect model
IV. Diseases of immune dysregulation	6	176	73(27–95)	< 0.01	83%	Random effects model
V. Congenital defects of phagocyte	8	184	39 (11–76)	< 0.01	80%	Random effects model
VII. Auto-inflammatory disorders	5	75	48 (10–88)	< 0.01	79%	Random effects model
Concerning geographical region						
Europe	14	692	68 (32–90)	< 0.01	92%	Random effects model
America	17	5432	57 (34–78)	< 0.01	97%	Random effects model
The Middle East	18	989	49 (20–79)	< 0.01	93%	Random effects model
Asia	9	334	41 (9–82)	< 0.01	82%	Random effects model

### Prevalence of Ocular Manifestations in IEI Patients According to the IUIS Classification

According to the IUIS classification, the prevalence for the first group, Immunodeficiencies affecting cellular and humoral immunity, stands at 15% (95%CI = 12–19, I2 = 0%, *p* = 0.55). The second group, CID with associated or Syndromic Features, showed a prevalence of 82% (95%CI = 66–91, I2 = 90%, p = < 0.01). For Predominantly Antibody deficiencies, it was 21% (95%CI = 17–26, I2 = 34%, *p* = 0.18), while, in Diseases of immune dysregulation, it was 73% (95%CI = 27–95, I2 = 79%, p = < 0.01). Congenital defects of phagocytes had a prevalence of 39% (95%CI = 11–76, I2 = 80%, p = < 0.01). Complement deficiencies, Bone marrow failure syndromes, and Phenocopies of PID could not be meta-analyzed due to insufficient data. Lastly, for Auto-inflammatory disorders, the pooled prevalence of ocular involvement was 48% (95%CI = 10–88, I2 = 79%, p = < 0.01) (Table 2).

### Prevalence of Eye Compromise in Patients with IEI According to Geographical Region

In the subgroup analysis by geographical regions, the prevalence of ocular manifestations in IEI was 68% in Europe (95%CI = 32–90, I2 = 92%, p = < 0.01), 57% in the Americas (95%CI = 34–78, I2 = 97%, p = < 0.01), 49% in the Middle East (95%CI = 20–79, I2 = 93%, p = < 0.01), and 41% in Asia (95%CI = 9–82, I2 = 82%, p = < 0.01), all derived using a random effects model and detailed in Table 2.

## Discussion

This meta-analysis reports the pooled prevalence of ocular involvement in patients with IEI from the data of 7,555 patients with IEI obtained from 62 studies published from 1968 to 2022. Ocular manifestations are reported in 54% of these patients (95%CI = 39–69, I2 = 94%). Nevertheless, it was challenging to establish definitive ocular conditions due to the variable methodology and low-moderate quality of the included studies. The variation of prevalence between studies was wide. Tunakan Dalgiç C [21, 33, 35, 37, 40, 42, 43, 45, 57, 58, 61, 64, 66–68, 70, 74]. This substantial variation can be attributed to differences in study designs and the population examined. Case series often record a heightened prevalence of ocular afflictions in IEI.

Despite the wide range of reported prevalences, our findings underscore that approximately half of the patients with IEI might exhibit some form of ocular manifestation, constituting a higher prevalence than those reported using methodologies similar to our study for conditions such as autoimmunity [77], or bronchiectasis [78], which are widely recognized as red-flags for suspecting IEI [79]; highlighting the significance of ophthalmological evaluations for individuals with IEI.

This study noted a male predominance, accounting for 59% of patients (95%CI = 55–63, I2 = 35%). Most of the literature supports a male predominance [80–84], often associated with the X-linked inheritance pattern of many IEI [80]. This male predominance occurs mainly in childhood stages; however, as the population ages, there is a change [83, 84], with a female predominance since X-linked inheritance diseases are less common [85]. Regarding ocular manifestations, Pham et al. reported that males had a 1.3 times greater risk of IEI with ocular manifestations than females [7].

A plethora of ocular manifestations were documented. These encompassed disturbances in ocular motility, notably nystagmus, strabismus, and oculomotor apraxia. Additionally, findings in the anterior segment were observed, including cataracts, ocular telangiectasia, and corneal opacity. Manifestations within the posterior segment, such as optic nerve disorders, chorioretinal scars, retinitis, and macular edema, were also noted. Each of these conditions induces varying degrees of visual impairment. Specifically, when the mean accumulative age was 11.1 ± 7.80 years, strabismus and cataracts heighten the risk of amblyopia [86]. Therefore, we hypothesized that patients with IEI and ocular involvement exhibit an increased amblyopia risk. This emphasizes the imperative nature of ophthalmological evaluations, especially during the pediatric age.

Patients with IEI have a higher susceptibility to both infectious and autoimmune manifestations [87, 88]. Concerning ocular conditions, Pham M et al. indicated that infectious ocular manifestations were more common than their noninfectious counterparts, with 64.7% and 40.3% prevalence, respectively [7]. A detailed meta-analysis breaking down the prevalence for specific manifestations was not undertaken in our study. However, we observed infectious manifestations such as conjunctivitis, keratitis, and hordeolum. Evaluation of ocular polyautoimmunity and coinfections in patients with IEI remains unexplored, necessitating future longitudinal studies.

Regarding the 2022 IUIS classification [3], the group of IEI most frequently linked to ocular manifestations, was CID associated with syndromic features, with a prevalence of 82% (95%CI = 66–91, I2 = 90%). This elevated prevalence might be explained by the significance of ophthalmological symptoms in the diagnostic criteria of specific diseases within this category. For instance, in Ataxia-Telangiectasia (AT), ocular telangiectasias and disturbances in eye movements [89, 90]. The European Society for Immunodeficiencies (ESID) regards these findings as indicative criteria for a probable AT diagnosis, which likely results in heightened clinical awareness and increased reporting of these manifestations than other diseases not traditionally associated with ocular presentations [91].

Europe was identified as the continent with the highest prevalence of IEI patients with ocular involvement, registering a rate of 68% (95%CI = 32–90, I2 = 92%). America (specifically the U.S.) followed this with a rate of 57% (95%CI = 34–78, I2 = 97%). This distribution is closely linked with IEI publications distribution, in which Europe and North America have the most significant number of published studies on this topic and two of the largest registries of IEI: USIDNET [92] and ESID [93]. This geographical pattern may also be explained by ethnic disparities, with individuals of caucasian ancestry being at higher risk compared to individuals of other ethnic backgrounds [7]. But also by socioeconomic factors in which limited resources in different regions lead to underestimation of the true prevalence [94].

The limitations of this study encompass selection, confounding, and interpretation biases, mainly because most of the included studies were observational case series and cross-sectional studies with an unclear risk of bias. Given these, it remains unclear what specific ocular manifestations ophthalmologists, pediatricians, and immunologists should look for about specific IEI. Longitudinal studies are needed to assess ophthalmological manifestations in specific IEIs across different age groups. Moreover, subgroup analyses were conducted to address this issue based on IEI groups and geographical regions, considering the anticipated substantial heterogeneity between studies due to their observational nature. While these subgroup analyses do not entirely mitigate the risks and the anticipated heterogeneity for prevalence meta-analysis, they provide a standardized approach to grouping studies and pinpointing differences tied to specific conditions [95–98]. In addition, since the inception of the IUIS-IEI classification in the 1970s, there have been considerable changes in the classification of immunodeficiencies, increasing the possibility of misclassification. However, for this study, diseases were categorized using the best clinical criteria available at the time. As previously stated, the quality of evidence is limited, as only observational studies are available, where the literature predominantly comprises cross-sectional and case series studies. Another limitation is the methodological bias in selected studies, with most case series having a high risk of bias and most remaining studies having an unclear risk of bias. Nonetheless, our study describes the pooled prevalence of ocular involvement in 7,555 patients with different clinical phenotypes of IEI. It shows the most extensive comprehensive characterization of ocular manifestations in IEI patients. Another limitation of our study was that we did not undertake a specific meta-analysis of the proportion of each ocular manifestation in IEI. However, the description of the number of articles on each manifestation can, in a certain way, characterize the ophthalmological manifestations in patients with IEI.

In summary, this study is the most extensive evaluation of ocular involvement in Inborn Errors of Immunity. Remarkably, our findings show that 54% of patients with IEI exhibit some ocular manifestation, with males being more affected, especially during childhood. These results highlight the imperative of improving the awareness among ophthalmologists, pediatricians, internists, and immunologists about the ocular manifestations linked to IEI, which will help prevent ocular complications such as amblyopia and improve these patients’ overall quality of life. Additionally, given the high documented prevalence, the importance of linking ophthalmologists with interdisciplinary teams for the care and follow-up of patients with IEI is emphasized. Further prospective cohort studies are required to look at the onset of ocular manifestations after diagnosing IEI or vice-versa for an opportune identification and treatment of ocular manifestations and IEI.

## Electronic Supplementary Material

Below is the link to the electronic supplementary material.

Supplementary Material 1

## Data Availability

No datasets were generated or analysed during the current study.
