# Supplementary material for: Prevalence of Ophthalmological Manifestations in Patients with Inborn Errors of Immunity: A Systematic Review and Meta-Analysis
Source: J Clin Immunol. 2025 May 13;45(1):92. doi: 10.1007/s10875-025-01880-4 (PMC12075347; doi:10.1007/s10875-025-01880-4)
Supplement: Supplementary file 1 — Supplementary Material 1 [file 10875_2025_1880_MOESM1_ESM.pdf]

Supplementary Data

1. **Supplementary methods.**

Meta-analysis of Observational Studies in Epidemiology (MOOSE) and 'Preferred Reporting Items for Systematic Review and Meta-analysis 'PRISMA, chart.

Reporting Criteria	Reported (Yes/No)	Reported on Page No.
**Reporting of Background**		
Problem definition	Yes	3
Hypothesis statement	Yes	3
Description of Study Outcome(s)	Yes	3
Type of exposure or intervention used	No	
Type of study design used	Yes	3
Study population	Yes	3
**Reporting of Search Strategy**		
Qualifications of searchers (eg, librarians and investigators)	No	
Search strategy, including time period included in the synthesis and keywords	Yes	4
Effort to include all available studies, including contact with authors	Yes	4
Databases and registries searched	Yes	4
Search software used, name and version, including special features used (eg, explosion)	Yes	4
Use of hand searching (eg, reference lists of obtained articles)	Yes	4
List of citations located and those excluded, including justification	Yes	4-5
Method for addressing articles published in languages other than English	No	
Method of handling abstracts and unpublished studies	Yes	4-5
Description of any contact with authors	No	
**Reporting of Methods**		
Description of relevance or appropriateness of studies assembled for assessing the hypothesis to be tested	Yes	4-5
Rationale for the selection and coding of data (eg, sound clinical principles or convenience)	Yes	5
Documentation of how data were classified and coded (eg, multiple raters, blinding, and interrater reliability)	Yes	5
Assessment of confounding (eg, comparability of cases and controls in studies where appropriate	No	

Reporting Criteria	Reported (Yes/No)	Reported on Page No.
Assessment of study quality, including blinding of quality assessors; stratification or regression on possible predictors of study results	Yes	5-6
Assessment of heterogeneity	Yes	7
Description of statistical methods (eg, complete description of fixed or random effects models, justification of whether the chosen models account for predictors of study results, dose-response models, or cumulative meta-analysis) in sufficient detail to be replicated	Yes	7
Provision of appropriate tables and graphics	Yes	24-34
**Reporting of Results**		
Table giving descriptive information for each study included	Yes	24-30
Results of sensitivity testing (eg, subgroup analysis)	Yes	11
Indication of statistical uncertainty of findings	Yes	8-11
**Reporting of Discussion**		
Quantitative assessment of bias (eg, publication bias)	Yes	13-14
Justification for exclusion (eg, exclusion of non–English-language citations)	No	
Assessment of quality of included studies	Yes	14
**Reporting of Conclusions**		
Consideration of alternative explanations for observed results	Yes	12-15
Generalization of the conclusions (ie, appropriate for the data presented and within the domain of the literature review)	Yes	15
Guidelines for future research	Yes	12-15
Disclosure of funding source	Yes	15

Search strategy

SEARCH STRATEGY PUBMED – DATE 21 MARCH 2022

Search	Search
Search Strategy Results n=2510	(("genetic diseases, inborn"[MeSH Terms] AND "Immunologic Deficiency Syndromes"[MeSH Terms]) OR ("Congenital Abnormalities"[MeSH Terms] AND "Immune System Diseases"[MeSH Terms]) OR "Primary Immunodeficiency Diseases"[MeSH Terms]) AND ("Eye Diseases"[MeSH Terms] OR "Eye Manifestations"[MeSH Terms] OR "Eye Abnormalities"[MeSH Terms] OR "Eye Infections"[MeSH Terms] OR "eye infections, bacterial"[MeSH Terms] OR "eye infections, fungal"[MeSH Terms] OR "eye infections, parasitic"[MeSH Terms] OR "eye infections, viral"[MeSH Terms] OR ("Uveitis"[MeSH Terms] OR "uveitis, intermediate"[MeSH Terms] OR "uveitis, posterior"[MeSH Terms] OR "uveitis, suppurative"[MeSH Terms] OR "uveitis, anterior"[MeSH Terms]))

SEARCH STRATEGY EMBASE– DATE 21 MARCH 2022

Search	Search
Search Strategy Results n=3731	(('immune deficiency'/exp OR 'deficiency, immune' OR 'immune defect' OR 'immune deficiency' OR 'immune deficiency disease' OR 'immune deficiency syndrome' OR 'immune deficit' OR 'immune depression' OR 'immune incompetence' OR 'immune response depression' OR 'immunity deficiency' OR 'immuno deficiency disease' OR 'immuno deficiency syndrome' OR 'immunodeficiency' OR 'immunodeficiency disease' OR 'immunodeficiency syndrome' OR 'immunodepression' OR 'immunologic deficiency' OR 'immunologic deficiency disease' OR 'immunologic deficiency syndrome' OR 'immunologic deficiency syndromes' OR 'immunological deficiency' OR 'immunological deficiency disease' OR 'immunosuppression, passive' OR 'immunosuppression, pathology' OR 'pathology of immunosuppression' OR 'primary immunodeficiency' OR 'primary immunodeficiency diseases' OR 'secondary immunodeficiency') AND ('monogenic disorder'/exp OR 'mendelian disease' OR 'mendelian disorder' OR 'mendelian inheritance disease' OR 'mendelian inheritance disorder' OR 'mendelian inherited disease' OR 'mendelian inherited disorder' OR 'mendelian inherited syndrome' OR 'mendelian syndrome' OR 'mono-genic disease' OR 'mono-genic disorder' OR 'monogene disease' OR 'monogene disorder' OR 'monogenetic disease' OR 'monogenetic disorder' OR 'monogenetic syndrome' OR 'monogenic disease' OR 'monogenic disorder' OR 'monogenic syndrome' OR 'single gene disease' OR 'single gene disorder' OR 'single gene syndrome') OR 'primary immunodeficiency disease'/exp OR 'primary immunodeficiency disorder'/exp OR 'primary immune deficiency'/exp OR 'inborn errors of immunity'/exp) AND ('eye disease'/exp OR 'disease, eye' OR 'eye disease' OR 'eye diseases' OR 'eye diseases, hereditary' OR 'eye disorder' OR 'eye manifestations' OR 'hereditary eye disease' OR 'hereditary eye diseases' OR 'neovascularisation, eye' OR 'neovascularization, eye' OR 'ocular abnormalities' OR 'ocular abnormality' OR 'ocular disease' OR 'ocular disorder' OR 'ocular disturbance' OR 'oculopathy' OR 'ophthalmic abnormality' OR 'ophthalmic disease' OR 'ophthalmic disorder' OR 'ophthalmic disturbance' OR 'ophthalmopathology' OR 'ophthalmopathy' OR 'eye inflammation'/exp OR 'eye inflammation' OR 'inflammation, eye' OR

	'ocular inflammation' OR 'ophthalmitis' OR 'eye infection'/exp OR 'eye infection' OR 'eye infections' OR 'intraocular infection' OR 'ocular infection')

SEARCH STRATEGY Virtual Health Library (VHL) – DATE 21 MARCH 2022

Search	Search
Search Strategy<br>Results<br>n=10,948<br>Those from MEDLINE are eliminated because we already have them with Pubmed and Embase, therefore there were 603.	(Primary Immunodeficiency Diseases) AND (Eye Diseases) OR (Eye Infections) OR (Uveitis)

Supplementary tables.

Table S1. Qualitative synthesis of ocular manifestations

Author, Year.	Country	Name of IEI	Patients with IEI	Patients with IEI and ocular manifestations	Type of ocular manifestation
Yaz I, et al. 2021 [46].	Turkey	Leukocyte adhesion deficiency Type I	15	1	Conjunctivitis
Azizi G, et al. 2020 [16].	Iran	Common variable immunodeficiency, hyper-IgM syndrome, agammaglobulinemia	310	50	Conjunctivitis
Yadav R, et al. 2020 [17].	India	Severe combined immune deficiency, Inborn errors of IFN-γ immunity, Combined immunodeficiency, Chronic granulomatous disease, Leucocyte Adhesion Deficiency type1.	52	1	Eye telangiectasias
Bistritzer J, et al.2021 [47].	Israel	Ataxia telangiectasia	15	2	Nystagmus
Deepti S, et al.2021 [18].	India	Deficiency of adenosine deaminase, STING-associated vasculopathy infantile-onset, Spondyloenchondro-dysplasia with Immune Dysregulation, Cryopyrin-Associated Periodic Syndromes, NLR Family, Pyrin domain-containing, Familial Mediterranean fever, Autoinflammation and PLCG2-associated antibody deficiency and immune dysregulation, TNF receptor-associated periodic syndrome, A20 haploinsufficiency, Laccase Domain Containing, Deficiency of Interleukin 1 Receptor Antagonist, TRNA nucleotidyl transferase, Pyogenic Arthritis, Pyoderma gangrenosum and Acne, Coatamer complex 1 protein alpha subunit syndrome, Caspase Recruitment Domain Family Member.	44	6	Ocular inflammation, opacity in the eye, eye swelling, conjunctivitis, blurred vision, papilledema, optic atrophy, corneal epithelial defect with corneal ulcers and corneal opacity.
Sukaiti N, et al. 2021 [19].	Australia	Severe combined immune deficiency	36	4	Eye infections (Klebsiella pneumonia, Haemophilus influenzae, Salmonella species)

Ferre E, et al. 2016 [20].	United States of America	Autoimmune polyendocrinopathy with candidiasis and ectodermal dystrophy	35	4	Keratoconjunctivitis
Ceyda Tunkan C, et al. 2017 [21].	Turkey	Primary immunodeficiency Disorders	92	1	Uveitis
Luo J, et al. 2021 [48].	China	Wiskott-Aldrich Syndrome	10	10	CMV retinitis
Boyarchuk O, et al. 2020 [22].	Ukraine	Ataxia telangiectasia	64	49	Oculomotor apraxia, nystagmus, eye telangiectasias
Al-Sulaiman R, et al. 2020 [49].	Qatar	Griscelli syndrome type 2	12	2	Strabismus
Yeh Y, et al. 2020 [71].	Taiwan	X-linked agammaglobulinemia	19	2	Deafness-dystonia-optic neuronopathy (DDON) syndrome
Barkai T, et al. 2020 [23].	Israel	Chronic granulomatous disease	16	1	Panuveitis
Qureshia S, et al. 2020 [50].	Pakistan	Severe combined immunodeficiency, Agammaglobulinemia/hypogammaglobulinemia, Wiskott-Aldrich Syndrome, Immunodeficiency Centromeric Instability and Facial Anomalies Syndrome, Hermansky-Pudlak Syndrome, Chronic granulomatous disease, Leukocyte adhesion deficiency, C3 deficiency	20	2	Oculocutaneous albinism

Massaad M, et al. 2020 [24].	Kuwait	C3 deficiency, RAG-1 deficiency, RAG-2 deficiency, Artemis deficiency, RFXANK-mediated MHC II deficiency, ICOS deficiency, DOCK2 deficiency, DOCK8 deficiency, Wiskott–Aldrich syndrome, DiGeorge syndrome, STAT5b deficiency, ICF syndrome with no documented genetic defect, AID deficiency, NF-kB2 deficiency , Selective IgA deficiency, CVID with no documented genetic defect, Chediak-Higashi syndrome, Fas deficiency, FasL deficiency, LRBA deficiency, APECED, IL-10 deficiency, CYBA deficiency, NCF2 deficiency, Blau syndrome, C4 deficiency	57	6	Uveitis, retinitis, chorioiriditis, iriditis
Faruk Incecik F, et al. 2020 [51].	Turkey	Ataxia telangiectasia	31	2	Oculomotor apraxia
Marques I, et al. 2019 [52].	Brazil	Chediak Higashi syndrome	14	4	Ocular albinism, decreased retinal pigmentation, nystagmus
Papadopoulou C, et al. 2019 [53].	Multicentric	Chronic granulomatous disease, Chronic mucocutaneous candidiasis, A20 haploinsufficiency, Periodic fevers with immunodeficiency and thrombocytopenia	10	3	Uveitis, conjunctivitis
Esenboga S, et al. 2017 [25].	Turkey	XL-agammaglobulinemia	32	2	Conjunctivitis
Lodice A, et al. 2017 [26].	Italy	Ataxia telangiectasia	15	15	Refractive errors, strabismus, fixation abnormalities, saccadic impairment, abnormal smooth pursuit, abnormal ocular movements
Mariani L, et al. 2017 [72].	France	Ataxia telangiectasia	17	10	Nystagmus
Akturk H, et al. 2017 [27].	Turkey	Ataxia telangiectasia	91	84	Oculocutaneous telangiectasia, nystagmus
Coulter T, et al. 2017 [28].	Non declared	Activated phosphoinositide 3-kinase delta syndrome	53	13	Conjunctivitis, orbital cellulitis, dacryocystitis, herpetic keratitis

Blazing S, eat al. 2016 [29].	Slovenia	Autoimmune lymphoproliferative syndrome, Activated phosphoinositide 3-kinase delta deficiency, Autoimmune polyendocrinopathy candidiasis ectodermal dysplasia, Ataxia telangiectasia, Deficiency of C2 component of complement, Common variable immunodeficiency, Immunoglobulin A deficiency, Lipopolysaccharide responsive beige-like anchor deficiency, Mucosa-associated lymphoid tissue lymphoma translocation 1 deficiency, Shwachman-Diamond syndrome, Severe combined immunodeficiency	247	5	Keratoconjunctivitis, allergic rhinoconjunctivitis
Nanthapisal S, et al. 2016 [30].	England	Adenosine deaminase 2 deficiency	15	1	Optic nerve atrophy
Patirogluh T, et al. 2016 [54].	Turkey	Oculocutaneous albinism	20	11	Nystagmus, lack of pigmentation in the iris and funds, atrophic changes of the peripheral retina, strabismus
Salman M.S, et al. 2015 [31].	Canada	Ataxia telangiectasia	184	115	Reduced visual acuity, optic disc abnormality, pupillary examination abnormality, visual field abnormality, strabismus, gaze-evoked nystagmus, impaired smooth pursuit, hypometric saccades, hypermetric saccades, impaired ductions
Méneret A, et al. 2014 [73].	France	Ataxia telangiectasia	67	13	Dysmetric saccades, eye telangiectasia, ocular movements abnormalities
Nagai K., et al. 2013 [55].	Japan	Chediak-higashi syndrome	15	14	Oculocutaneous albinism.
Greenberger S, et al. 2013 [32].	Israel	Ataxia telangiectasia	32	31	Ocular telangiectasia (interpalpebral bulbar conjunctiva), pigmentary anomalies included cafe-au-lait macules, hypopigmented macules, and melanocytic nevi, oculocutaneous albinism

Malgorzara P, et al. 2013 [56].	Poland	X-linked agammaglobulinemia	33	6	Conjunctivitis
Shaikh A, et al. 2010 [74].	Switzerland, United States of America	Ataxia telangiectasia	13	13	Horizontal gaze-evoked nystagmus in primary gaze, vertical nystagmus, impaired visual suppression
Alaaeldin F, et al. 2010 [33].	Egypt	Chediak–Higashi syndrome, Hermansky–Pudlak syndrome, Oculocutaneous albinism	113	113	Oculocutaneous albinism
Tsilou E, et al. 2010 [34].	United States of America	Dyskeratosis congenita, Fanconi anemia	50	31	Cataracts, palpebral epicanthus, ptosis, limbal neovascularization, embryotoxon, anomalies of the lacrimal drainage system (absence of punctum, nasolacrimal obstruction), scar entropion, keratoconjunctivitis, exudative retinopathy, microcornea, microphthalmia, myopia, astigmatism, granular retinal pigment epithelial changes, retinal detachment, vascular sheathing
Shaikh A, et al. 2009 [35].	United States of America	Ataxia telangiectasia	13	13	Nystagmus, sadistic movements (microsaccade oscillations and square wave saccadic intrusions)
Al-Muhsen S, et al. 2009 [76].	Saudi Arabia	Chronic Granulomatous Disease	32	14	Seborrheic blepharitis, stye, episcleritis, bilateral active anterior uveitis with posterior synchiae, ring-shaped corneal scarring of the inferior stroma, chorioretinal lesions
Khan A, et al. 2008 [57].	Saudi Arabia	Ataxia telangiectasia	11	11	Saccadic dysfunction, delayed convergence, abolished convergence, exotropia, gaze-evoked nystagmus, downbeat nystagmus
Nofech-Mozes Y, et al. 2007 [36].	Canada	Adenosine deaminase deficiency	14	2	Rotary nystagmus, eye rolling

Riise R, et al. 2007 [58].	Norway	Ataxia telangiectasia	10	10	Convergent strabismus, photophobia, loss of fixation, nystagmus, abnormal saccades, conjunctival telangiectasias
Moin M, et al. 2007 [37].	Iran	Ataxia telangiectasia	104	104	Apraxia of horizontal and vertical saccadic eye movements, conjunctival telangiectasia
Winkelstein J, et al. 2006 [38].	United States of America (multicentric)	X-linked agammaglobulinemia	201	42	Conjunctivitis
Rezaei N, et al. 2005 [59].	Iran	Cyclic neutropenia, Shwachman-Diamond syndrome, Kostmann syndrome, Chediak-Higashi syndrome	26	2	Conjunctivitis
Farr A, et al. 2002 [39].	United States of America	Ataxia telangiectasia	63	57	Strabismus (endodeviation, exodeviation, hyperdeviation), conjunctival telangiectasia, myopia, hyperopia, nystagmus, optokinetic nystagmus, saccadic delay, Jerky pursuit
Kivitie-Kallio S, et al. 2001 [40].	Finland	Cohen syndrome	29	29	Antimongoloid slant of the eyelids, high-arched or wavy eyelids, long/thick eyelashes, thick eyebrows, retinitis pigmentosa-like retinal dystrophy, and progressive myopia
Boerkoel C, et al. 2000 [41].	Canada	Schimke immuno-osseous dysplasia	39	7	Corneal opacity, myopia and astigmatism, bilateral optic atrophy
Kawame H, et al. 1998 [60].	United States of America	Kabuki syndrome	18	11	Leukoma, eyelid ptosis, Marcus Gunn's phenomenon, strabismus, mild bilateral hypoplasia of the opic nerve, cataracts

Lewis R, et al. 1999 [61].	United States of America	Ataxia telangiectasia	56	56	Spontaneous Nystagmus, Saccades, Slow vestibulo-ocular reflex, Slow Optokinetic response, Centrifugal diffraction in gaze retention, esotropia, impaired convergence
Rudge P, et al. 1996 [62].	England	X-Linked Agammaglobulinemia, Common Variable Immunodeficiency Phenotype	13	5	Nystagmus, chorioretinitis, restriction of eye movements, transient blurred vision, papilledema, retinitis pigmentosa, decreased visual acuity, constriction of visual fields
Ziv Y, et al. 1992 [42].	Israel	Ataxia telangiectasia	19	19	Congenital cataract, nystagmus
Palestine A, et al. 1983 [63].	United States of America	Neutrophil dysfunction	32	9	Keratitis, chorioretinal scars, blepharoconjuntivits
Latkany P, et al. 2002 [64].	United States of America	Systemic granulomatosis	16	16	Anterior uveitis, posterior uveitis, cataracts, glaucoma, multifocal choroiditis
Woods C, et al. 1992 [43].	British islands	Ataxia telangiectasia	70	70	Oculomotor apraxia
Goldblatt D, et al. 1999 [75].	England	Chronic granulomatous disease	38	9	Chorioretinal lesions
Alyasin S, et al. 2019 [65].	Iran	Ataxia telangiectasia	18	13	Oculocutaneous telangiectasia, oculomotor apraxia
Cohen L, et al. 1984 [66].	United States of America	Ataxia telangiectasia	12	12	Conjunctival telangiectasias, hypopigmented macula
Farina L, et al. 1994 [67].	Italy	Ataxia telangiectasia	12	12	Nystagmus, oculomotor apraxia, oculocutaneous telangiectasias

JaY B, et al. 1968 [68].	England	Ichthyosis	62	62	Ectropion, ichthyosis of the eyelids vulgari, thickening of the conjunctiva, corneal changes, superficial punctate keratitis, stromal changes, lenticular alterations, peripheral granular pigmentation, Marcus Gunn phenomenon
Kim S, et al. 2003 [69].	South Korea	Chronic granulomatous disease	17	6	Chorioretinal lesions, RPE atrophy, chorioretinal atrophy, vision loss, peripheral retinal ischemia, neovascular membrane, macular edema
Veerapandiyan A, et al. 2011 [44].	United States of America (multicentric)	DiGeorge syndrome	50	20	Short palpebral fissures, hypertelorism, epicanthal folds, hooded eyelids, upslanting palpebral fissures, downslanting palpebral fissures, and ptosis.
Pham M, et al. 2022 [7].	Multicentric (United States of America and Canada)	Immunodeficiencies affecting cellular and humoral immunity, CID with associated or syndromic features, Predominantly antibody deficiencies, Immune dysregulation, Defects in phagocytosis, Defects in intrinsic and innate immunity, Autoinflammatory disorders, Complement deficiencies, Phenocopies of inborn errors of immunity, and Unknown or undetermined immunodeficiency	4624	519	Conjunctivitis, blepharitis, stye, chalazion, corneal infection, chorioretinitis, endophthalmitis, keratoconjunctivitis, ocular VZV, vitritis, episcleritis, infected eye prosthesis, orbital cellulitis, choroidal mass<br>Decreased vision, uveitis, photophobia, diseases of the retina, cataracts, glaucoma, optic nerve disease, corneal disease, eye movement disorders, ptosis, strabismus, dry eye
Manjunath M, et al. 2020 [45].	India	Ataxia telangiectasia	100	100	Ocular telangiectasia, ocular apraxia, impaired pursuit and slow saccades, nystagmus

Huryn L, et al. 2022 [70].	United States of America	Retinal dystrophy, optic nerve edema, splenomegalia, anhidrosis and headache	11	11	Retinal dystrophy, optic nerve edema, anhidrosis, cystoid macular edema

IgM: Immunoglobulin M, IFN-γ: Interferon-gamma, STING: Stimulator of Interferon Genes, NLR: Nod-Like receptor, TNF: Tumor necrosis factor, C3: Complement component 3, RAG-1: Recombination-activating gene 1, RAG-2: Recombination-activating gene 2, RFXANK: RFXAP-containing ankyrin repeat, MHC-II: Major histocompatibility complex type II, ICOS: Inducible costimulator, DOCK2: Dedicator of cytokinesis 2, DOCK8: Dedicator of cytokinesis 8, STAT5b: Signal transducer and activator of transcription 5B, ICF: Immunodeficiency with centromeric instability and facial anomalies, AID: Activation-Induced Cytidine Deaminase, NF-kB2: Nuclear factor kappa-B subunit 2 gene, IgA: Immunoglobulin A, CVID: Common variable immunodeficiency, LRBA: Lipopolysaccharide-responsive beige-like anchor protein, APECED: Autoimmune polyendocrinopathy candidiasis ectodermal dystrophy, IL-10: Interleukin-10, NCF2: Neutrophil cytosolic factor 2, C4: Complement component 4, C2: Complement component 2, and XL: X-linked.

Supplementary figures legends.

Figure S1. Risk of bias traffic-light plot of cohort studies

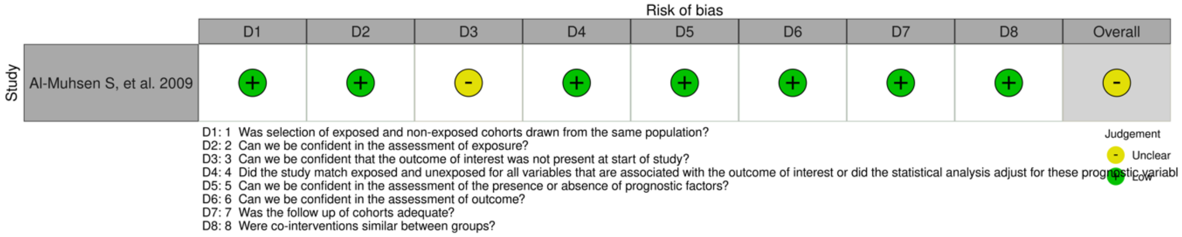

Domain scoring: Definitely yes (low); Probably yes or Probably no (some concern); Definitely no (High).

Figure S2. Risk of bias traffic-light plot of case-control studies.

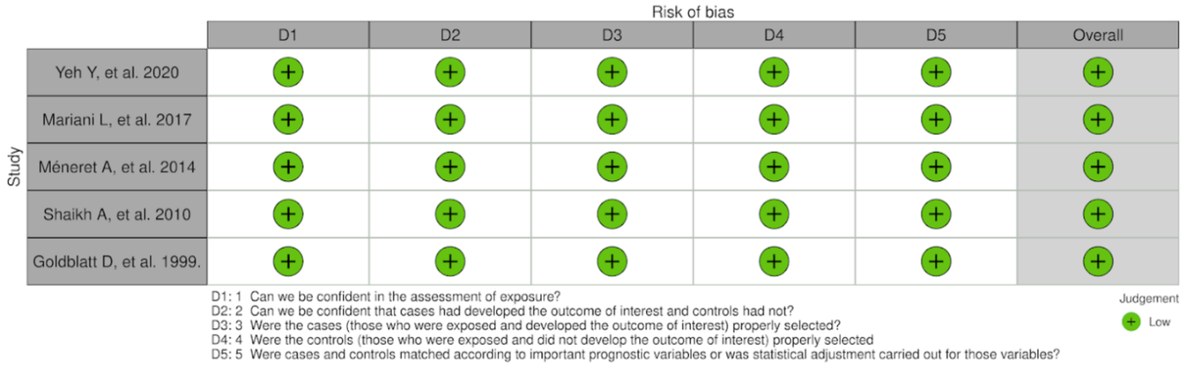

Domain scoring: Definitely yes (low); Probably yes or Probably no (some concern); Definitely no (High).

Figure S3. Risk of bias traffic-light plot of cross-sectional studies.

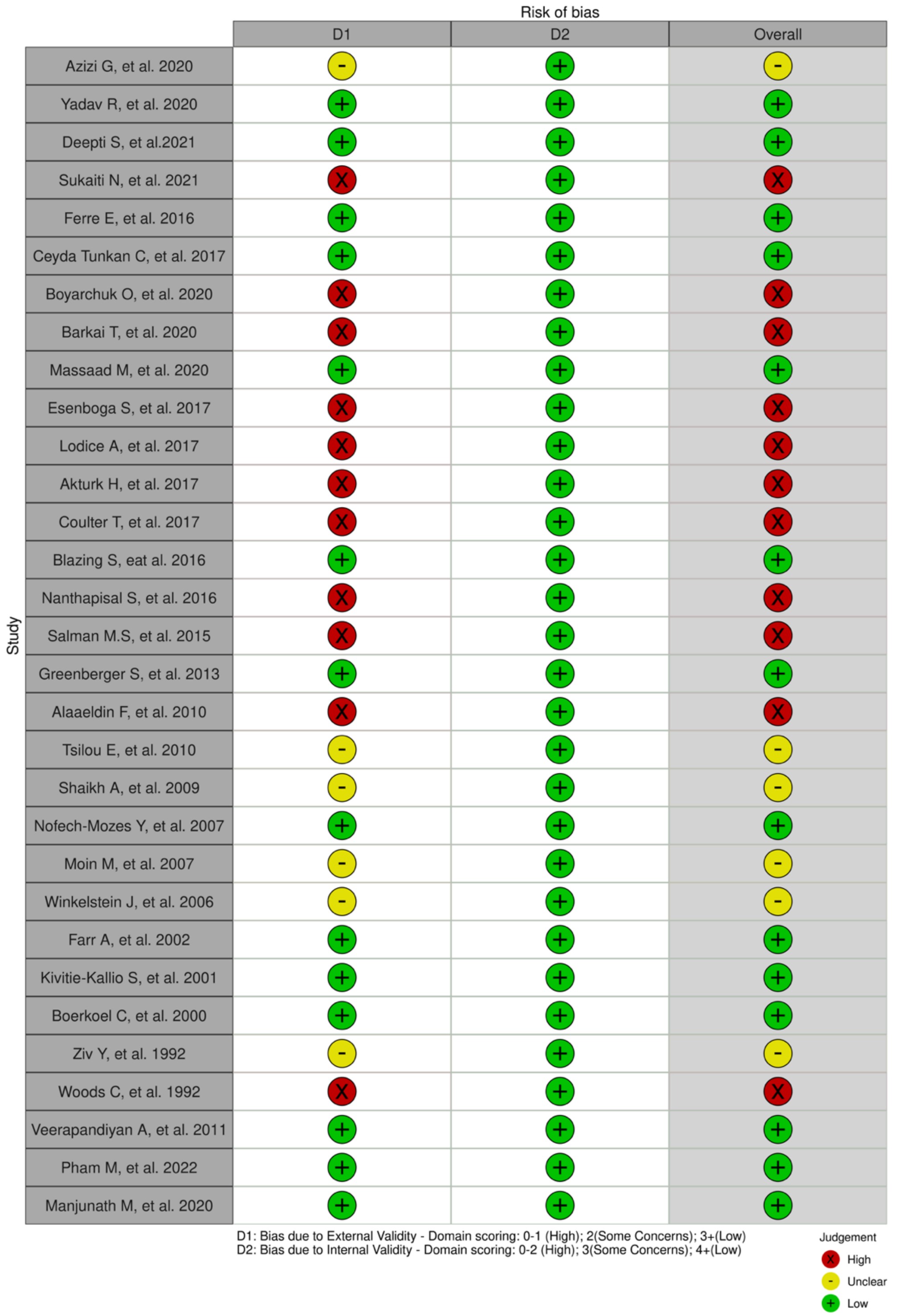

Domain scoring: External Validity - Domain scoring: 0-1 (High); 2(Some Concerns); 3+(Low). Internal Validity - Domain scoring: 0-2 (High); 3(Some Concerns); 4+(Low).

Figure S4. Risk of bias traffic-light plot of case-series studies.

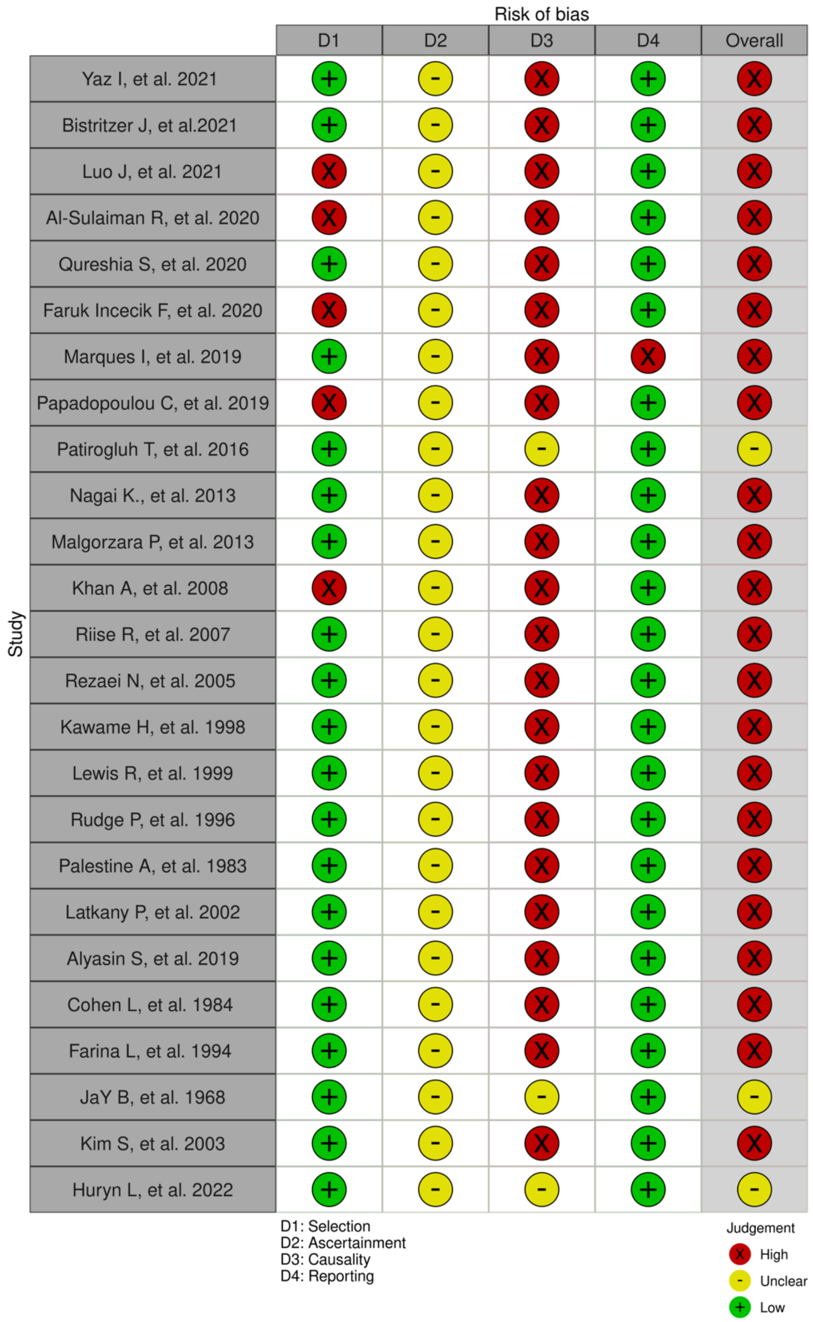

Domain scoring: Selection - Domain scoring: 0-1 (High); 2(Some Concerns); 4+(Low). Ascertainment - Domain scoring: 0-2 (High); 3(Some Concerns); 4+(Low). Causality - Domain scoring: 0-2 (High); 3(Some Concerns); 4+(Low). Reporting - Domain scoring: 0-2 (High); 3(Some Concerns); 4+(Low).
